# The heat is on: scaling improvements in photosynthetic thermal tolerance from the leaf to canopy to predict crop yields in a changing climate

**DOI:** 10.1098/rstb.2024.0235

**Published:** 2025-05-29

**Authors:** Amanda Cavanagh, Megan Matthews

**Affiliations:** ^1^ School of Life Science, University of Essex - Colchester Campus, Colchester, UK; ^2^ Carl R Woese Institute for Genomic Biology, University of Illinois Urbana-Champaign, Urbana, IL, USA; ^3^ Department of Civil and Environmental Engineering, Grainger College of Engineering, University of Illinois Urbana-Champaign, Urbana, IL, USA

**Keywords:** photosynthesis, temperature stress, photorespiration, climate change

## Abstract

Crop production must increase to sustain a growing global population, and this challenge is compounded by increased growing season temperatures and extreme heat events that are already causing significant yield losses in staple crops. Therefore, there is an urgent need to develop strategies to adapt crops to withstand the impacts of a warmer climate. Temperature-sensitive vegetative processes fundamentally related to yield, like photosynthesis, will be impacted by warming throughout the growing season, thus strategies to enhance their resilience hold promise to future-proof crops for a warmer world. Here, we summarize three major strategies to enhance C3 photosynthesis above the thermal optimum: enhanced rubisco activation, modified photorespiration and increased rates of ribulose bisphosphate regeneration. We highlight recent experimental evidence demonstrating the efficacy of these strategies, and then use a mechanistic modelling approach to predict the benefit of these engineering strategies on leaf-level carbon assimilation and soybean yield at elevated temperatures. Our approach highlights that these three engineering targets, particularly when combined, can enhance photosynthetic rates and yield under both ambient and elevated temperatures. By targeting multiple aspects of photosynthetic metabolism, we can develop crops that are better equipped to withstand the challenges of a warming climate and contribute to future food security.

This article is part of the theme issue ‘Crops under stress: can we mitigate the impacts of climate change on agriculture and launch the ‘Resilience Revolution’?’.

## 1. Introduction

By 2050, global food demand is projected to increase up to an additional 50% of current demand than it is today [1]. However, current trends in agricultural productivity make achieving this target incredibly difficult [2,3]. This challenge is intensified by climate change, which poses significant threats to crop yields worldwide. Terrestrial mean global temperatures have increased approximately 1.6°C since the industrial revolution [4], and current global policies and actions will probably result in an increase from 3.3 to 5.7°C in mean global temperature by the end of the century [4,5]. These rising temperatures have already led to global yield losses in staple crops like maize and wheat [6,7] and further increases are projected to negatively impact the yields of these and other essential crops, such as rice and soybean [8]. Even if we are to achieve zero carbon emissions immediately, the warming caused by the accumulation of atmospheric CO_2_ will persist owing to the cumulative effects of past emissions. Therefore, there is an urgent need to develop strategies that not only improve crop yields but also adapt crops to withstand the adverse effects of a warmer climate.

In the face of both growing global food demands and the pressures of climate change, improving photosynthetic efficiency has emerged as a key strategy to improve crop yields. Photosynthesis is the fundamental process driving plant growth and the production of food, fibre and biofuels and photosynthetic efficiency directly influences crop yields [9–12]. In C3 plants, net photosynthetic carbon assimilation (*A*_N_) is most often constrained by rubisco carboxylation or ribulose bisphosphate (RuBP) regeneration capacity [13]. Under current atmospheric [CO_2_] and saturating light, the temperature dependence of *A*_N_ is co-limited by declines in both rubisco biochemistry and chloroplast electron transport rates [14–16]. Owing to the direct impacts increased chloroplast electron transport, RuBP regeneration and rubisco biochemistry have on the temperature response of photosynthesis, all have been an important target for transgenic modification (table 1).

**Table 1.** Summary of validated targets demonstrating increased rubisco activation (RA), RuBP regeneration (RuBP) or lowered photorespiratory losses (PR) and their impact on photosynthesis and growth at ambient and warming conditions. (Rca = rubisco activase (EC 4.1.1.39); SBPase = sedoheptulose 1,7-bisphosphatase (EC 3.1.3.37); FBPaldolase = (EC 4.1.2.13); Cyt *c*_6_ = algal cytochrome *c*_6_; SBPase/FBPase = (EC3.1.3.37 + EC 3.1.3.11); GDC = glycine decarboxylase complex (EC 2.1.2.10**,** including subunits T, P, L and H); GlcDH = glycolate dehydrogenase (EC 1.1.99.14); GOX = glycolate oxidase (EC 1.1.3.15); WT = wild-type.)

strategy	target	species	impact on photosynthesis and growth
RA	enhanced thermotolerance of Rca	Arabidopsis	overexpression of thermostable Rca isoforms maintains increased rates of rubisco activation state, *A*_N_ and growth relative to WT under heat stress [17,18]
		rice	co-overexpression of thermostable Rca and rubisco increase *V*_cmax^′^_ *A*_N′_ and biomass relative to WT at 40°C [19]
RuBP	overexpression of SBPase	Arabidopsis, tobacco, tomato, wheat	increased *V*_cmax^′^_ *J*_max″_ *A*_N_ and biomass relative to WT under ambient conditions [20–23]; and increased seed and grain yield [20,24]. Increased *J*_max_ and growth relative to WT under elevated CO_2_ [25]
		rice	increased *A*_N_> 35°C, and is associated with higher growth rates compared with WT under elevated temperature [26]
	overexpression of FBPaldolase	Arabidopsis, tobacco, tomato	increased *V*_cmax′_ *J*_max**″**_ *A*_N_ and biomass relative to WT under ambient conditions [20]. Increases *A*_N_ in tomato [27]. Further increases in *A*_N_ and growth evident at elevated CO_2_ [28]
	overexpression of bifunctional SBPase/FBPase	tobacco, lettuce	increases in *A*_N_ and biomass relative to controls [29,30]
		soybean	increased *A*_N,_*V*_cmax′_ and *J*_max_ relative to WT. Protective against losses in seed yield loss only when grown under combined elevated temperature and CO_2_ field conditions [31]
	overexpression of algal Cyt *c*_6_	Arabidopsis, tobacco	increased *A*_N_ and plant growth [32,33] and water-use [33]
	overexpression of Rieske FeS protein	Arabidopsis	increases in *A*_max,_*J*_max′_ biomass and seed yield relative to WT controls [34]
		tobacco	transient increases in Cyt *b*_6_*f* activity, but no increases in *V*_cmax′_ *J*_max′_ or growth [35]
	combined overexpression of algal Cyt *c*_6_ with bifunctional SBPase/FBPase or SBPase	tobacco	increases in *J*_max,_*A*_max′_ biomass and water-use efficiency in-field conditions [36]
PR	chloroplast expression of alternative photorespiration pathways	Arabidopsis, Camelina, tobacco, potato, rice	fully decarboxylating pathways demonstrate decreases in CO_2_ compensation point [37–43], increases in *A*_N_ at supra-ambient temperatures [37,44]. All show environmental-dependent increases in *V*_cmax_ and *J*_max_ [38,39,41–43,45]. Growth stimulation, particularly under high light, high temperature conditions [37–44,46]
	overexpression of glycine decarboxylase (GDC) complex	Arabidopsis, tobacco	increased *A*_N_, particularly at high light [20,47,48]. Decreased CO_2_ compensation point [48,49]. Enhanced growth relative to WT controls, particularly under high light conditions
	overexpression of 2 PG phosphatase (PGLP1)	Arabidopsis	increased *A*_sat″_ photosynthetic and electron transport at elevated temperatures. Lower CO_2_ compensation point under prolonged heat stress or drought stress [50]
	overexpression of glycolate dehydrogenase (GlcDH)	Arabidopsis, potato, Camelina	decreases in CO_2_ compensation point [40,51], increases in *A*_N_ [45,51,52], *V*_cmax′_ *J*_max_ [45] and photosynthetic electron transport [52]. Biomass and yield stimulation
	overexpression of glycolate oxidase (GOX)	rice	increase in *A*_N_ compared with WT at high temperature and high light conditions. Lines with 60–100% more GOX expression demonstrate increased plant height under ambient conditions [53]

Rubisco is regulated by a heat-sensitive chaperone protein known as rubisco activase (Rca), which plays a critical role in removing inhibitory sugar-phosphate compounds from rubisco’s catalytic sites, thereby sustaining photosynthetic carboxylation efficiency [54–57]. Rubisco remains functional at temperatures exceeding 50°C; however, most Rca isoforms lose activity around 40°C [58,59]. Consequently, the *in vivo* deactivation of rubisco at elevated temperatures is linked to the thermal sensitivity of Rca. This, in turn, triggers photosynthetic inhibition, ultimately leading to yield reductions [56]. Many species have multiple isoforms of Rca with varying heat tolerance [60–62]. Transgenic overexpression of a thermostable Rca isoform confers improved photosynthetic performance and recovery after a short-term high-temperature exposure [17,63] and thermoprotective growth advantages at high growth temperatures [19].

Maintaining rubisco activation (RA) at higher temperatures may also promote rubisco oxygenation, and subsequent photorespiratory losses, although impacts of this remain relatively unexplored. Rubisco’s substrate specificity for CO_2_ also diminishes with rising temperatures, leading to an increase in RuBP oxygenation rather than carboxylation, and CO_2_ release through photorespiration (PR) [64–67]. PR salvages one molecule of 3-phosphoglycerate (3-PGA) from two molecules of the oxygenation product 2-phosphoglycolate (2 PG) with the release of one molecule of CO_2_ through a series of enzymatic conversions and transport steps spanning the chloroplast, peroxisome and mitochondria [68]. The photorespiratory pathway is energetically costly, requiring 3.5 ATP and 2 NADPH equivalents to recover RuBP from 2 PG and 3-PGA and releases NH3 and 25% of the previously fixed CO_2_ in the mitochondria. This can result in a dramatic yield drag in C3 crops, with modelled wheat yield losses of 20% across the USA annually and yield reductions of 50% or more observed in warmer growing regions [69]. Photorespiratory flux can be directly manipulated to enhance growth and photosynthesis in heated conditions through the introduction of synthetic 2 PG metabolic pathways (‘bypasses’) [37] and by the overexpression of key enzymes that may act to optimize photorespiratory flux [50].

As temperatures continue to rise, chloroplast electron transport and RuBP regeneration will become a limiting factor for *A*_N_ [15]. Additionally, elevated [CO_2_] levels are anticipated to shift the control of photosynthesis from rubisco to RuBP regeneration in C3 crops [70]. Transgenic rice plants with enhanced RuBP regeneration maintain higher rates of *A*_N_ after transient heat stress above 35°C [26]. In transgenic soybean, enhanced RuBP regeneration capacity conferred a thermoprotective benefit to in-field warming under elevated CO_2_ conditions [31]. However, this strategy has not been fully explored to improve thermotolerance, and mechanistic measurements into the impact of enhanced RuBP regeneration on photosynthetic temperature responses remain unclear.

A key feature of the many strategies to improve photosynthetic carbon gain above the thermal optima is the modularity of the approaches. Many singular targets for manipulation have been identified to enhance RA, increase RuBP regeneration, or lower the cost of PR (table 1). Stacking of improvements made from several targets together will avoid co-limitation by other processes. Multigene constructs targeting the co-overexpression of RuBP regeneration and optimized photorespiratory flux demonstrate that additive gains in photosynthesis and biomass in controlled conditions are possible [20]. However, strategies remain untested over a broad range of temperatures. In this work, we take advantage of mechanistic modelling of photosynthesis at the leaf and canopy level to present a predictive approach to identify synergies between three important targets used to enhance photosynthesis above the thermal optima.

## 2. Modelling modified photosynthesis above the thermal optimum

The impact of targeted manipulations on net photosynthetic carbon assimilation (*A*_N_) can be predicted using the mechanistic Farquhar von Caemmerer Berry (FvCB) model of photosynthesis [13,71]. The temperature response of light-saturated *A*_N_ can be modelled by the minimum of rubisco-limited photosynthesis (*A*_c_) and RuBP-limited photosynthesis (*A*_j_) as:


(2.1) $$A_{c}=\frac{\left(C-\Gamma^{*}\right)V_{cmax}}{C+K_{C}\left(1+O/K_{O}\right)}-R_{l},$$

(2.2) $$A_{j}=\;\frac{\left(C-\Gamma^{*}\right)J_{max}}{4C+8\Gamma^{*}}-R_{l},$$

where Γ* represents the CO_2_ compensation point, *V*_cmax_ is the maximum rate of rubisco carboxylation, *J*_max_ is the light-saturated electron transport rate, *O* and *C* are the oxygen and carbon dioxide concentrations, *K*_C_ and *K*_O_ are the Michaelis–Menten constants for CO_2_ and O_2_, respectively, and *R*_l_ represents the carbon dioxide release in the light independent of PR. Although assimilation can also be limited by the rate of triose phosphate utilization, this is primarily observed at saturating CO_2_ or low temperatures, and so we have ignored this limitation in our focus on high-temperature responses.

The temperature dependencies of the six parameters (Γ*, *V*_cmax′_ *J*_max_ *R*_d′_ *K*_C_ and *K*_O_) are described by the Arrhenius equation:


(2.3) $$parameter=exp\left(c-\Delta H_{a}/RT_{k}\;\right),$$

where *c* is the scaling constant, Δ*H*_a_ is the activation energy, *R* is the molar gas constant (8.314 J mol^−1^ K^−1^) and *T_k_* is the leaf temperature in Kelvin [72–74]. The mechanistic basis of enhanced or maintained *A*_N_ at increased temperature in plants with modified PR, increased RA or increased RuBP regeneration (RuBP) are likely to be related to changes in either the absolute value or the temperature responses of these six parameters. However, comparisons between improvement strategies are complicated because full temperature datasets on Γ*, *V*_cmax′_ and *J*_max_ are only available for tobacco plants expressing a synthetic glycolate metabolic pathway to divert photorespiratory flux [37]. In these plants, enhanced *A*_N_ above the thermal optima is probably driven by a change in the apparent Δ*H*_a_ for Γ* such that the parameter is lower in transgenic plants at temperatures above 35°C (table 2). Changes in *V*_cmax_ and *J*_max_ responses resulting from increased RA or enhanced electron transport can be mechanistically modelled from *in vitro* kinetic responses [16,75,76], but these may not always reflect the phenotype observed from *in planta* manipulations. In the absence of full temperature responses of these parameters for other manipulations, constants and Δ*H*_a_ of *V*_cmax_ and *J*_max_ can be extrapolated from experimental data to approximate the theoretical phenotype. Enhancing RA state through the overexpression of Rca has increased rice *V*_cmax_ in heated conditions but not controlled conditions [64], which can be modelled through altered Δ*H*_a_ (table 2). Enhanced RuBP regeneration/electron transport was modelled as a 15% increase in wild-type (WT) *J*_max_ at all temperatures (table 2), consistent with the upper range of reported increases in *J*_max_ when in plants expressing SBPase or Rieske FeS proteins [21,34,36], and experimental evidence finding no differential changes in the temperature response of *J*_max_ in transgenic plants with enhanced *J*_max_ from the overexpression of a bifunctional SBPase/FBPase [31]. The temperature response of *A*_N_ in unmodified plants was modelled using the minimum of equations (2.1) and (2.2), using the temperature responses of *K*_C_ and *K*_O_ from [74] and WT values of *V*_cmax_ and *J*_max_ from [37]. The temperature response of rubisco kinetic parameters (i.e. *K*_C_ and *K*_O_) are available *in vivo* from *Nicotiana tabacum* (tobacco) and *Arabidopsis* [72,73,77], but not *Glycine max*. To minimize errors in estimating photosynthetic CO_2_ assimilation without these key species-specific parameters [78,79] and enable stronger comparison with experimentally derived data in a modelled crop [21,36,37], leaf-level responses are modelled in tobacco. Parameters used to model temperature responses are given in table 2.

**Table 2.** Parameters used to calculate the temperature response of *A*_N_ including the activation energy (ΔH_a_) and scaling constant (*c*). Representative values for unmodified and modified PR plants are from [37]; altered parameters are based on experimental evidence for manipulated Rca [19] (RA) and RuBP regeneration [21,31] (RuBP) as described in text.

	unmodified		RA		PR		RuBP	
parameter	*c*	*ΔH*_a_	*c*	*ΔH*_a_	*c*	*ΔH*_a_	*c*	*ΔH*_a_
*V*_cmax_	27.68	57.96	29.7	62.88				
*J*_max_	21.6	41.76					21.78	41.76
Γ* (Pa)	15.6	34.8			13.3	29.2		

## 3. Photosynthesis above the thermal optimum

**Figure 1.**
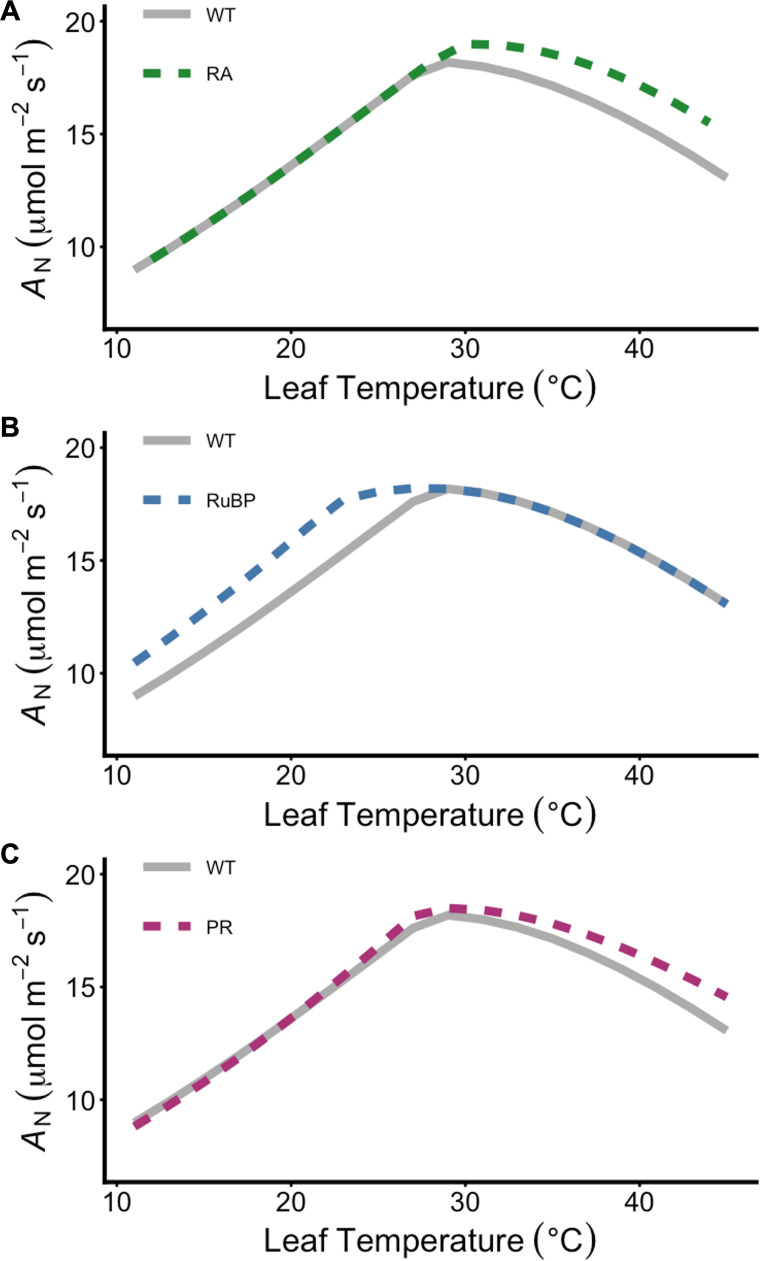
Predicted impacts of engineering strategies at and above the thermal optima. The temperature response of net photosynthetic CO_2_ assimilation rate (*A*_N_) predicted as the minimum of carboxylation-limited photosynthesis (*A*_C_) or RuBP-regenerated photosynthesis (*A*_j_) based on chloroplast electron transport. Modelled outputs were obtained at 21% O_2_ and light-saturated conditions (i.e. 1500 μmol m^−2^s^−1^) assuming an ambient CO_2_ concentration of 410 ppm using parameters described in table 2. (A) The response of enhanced RA through rubisco activase (Rca) overexpression (RA; dashed green lines) compared with unmodified plants (WT; solid grey lines). (B) The response of enhanced electron transport or RuBP regeneration rates (RuBP; dashed blue lines) compared with unmodified plants (WT; solid grey lines). (C) The response of modified PR (dashed purple lines) compared with unmodified plants (WT; solid grey lines).

When modelled over a temperature range of 10–45°C, strategies that increase RA or modify PR have similar rates of *A*_N_ at temperatures leading up to peak photosynthetic rates (figure 1A–C). Maintaining higher rates of *V*_cmax_ through enhanced RA increases both the maximum photosynthetic rate (*A*_max_) and the temperature associated with maximum *A*_N_ (*T*_max_). Further increases in *A*_N_ of 5–12% are predicted at temperatures above *T*_max_ (figure 1A), in line with empirical work demonstrating a benefit to the expression of a thermostable variant of Rca [19,61,63]. The thermal optimum is unchanged by modified PR, but *A*_max_ is approximately 2% higher than WT levels, and at temperatures above approximately 30°C, where rubisco more strongly favours oxygenation over carboxylation, modified PR enhances *A*_N_, maintaining rates that are approximately 7% greater than unmodified rates at 40°C (figure 1C). Unsurprisingly, modelled impacts of increased electron transport demonstrate increased *A*_N_ only below *T*_max′_ where rates of *A*_j_ are the predominant limitation (figure 1B).

**Figure 2.**
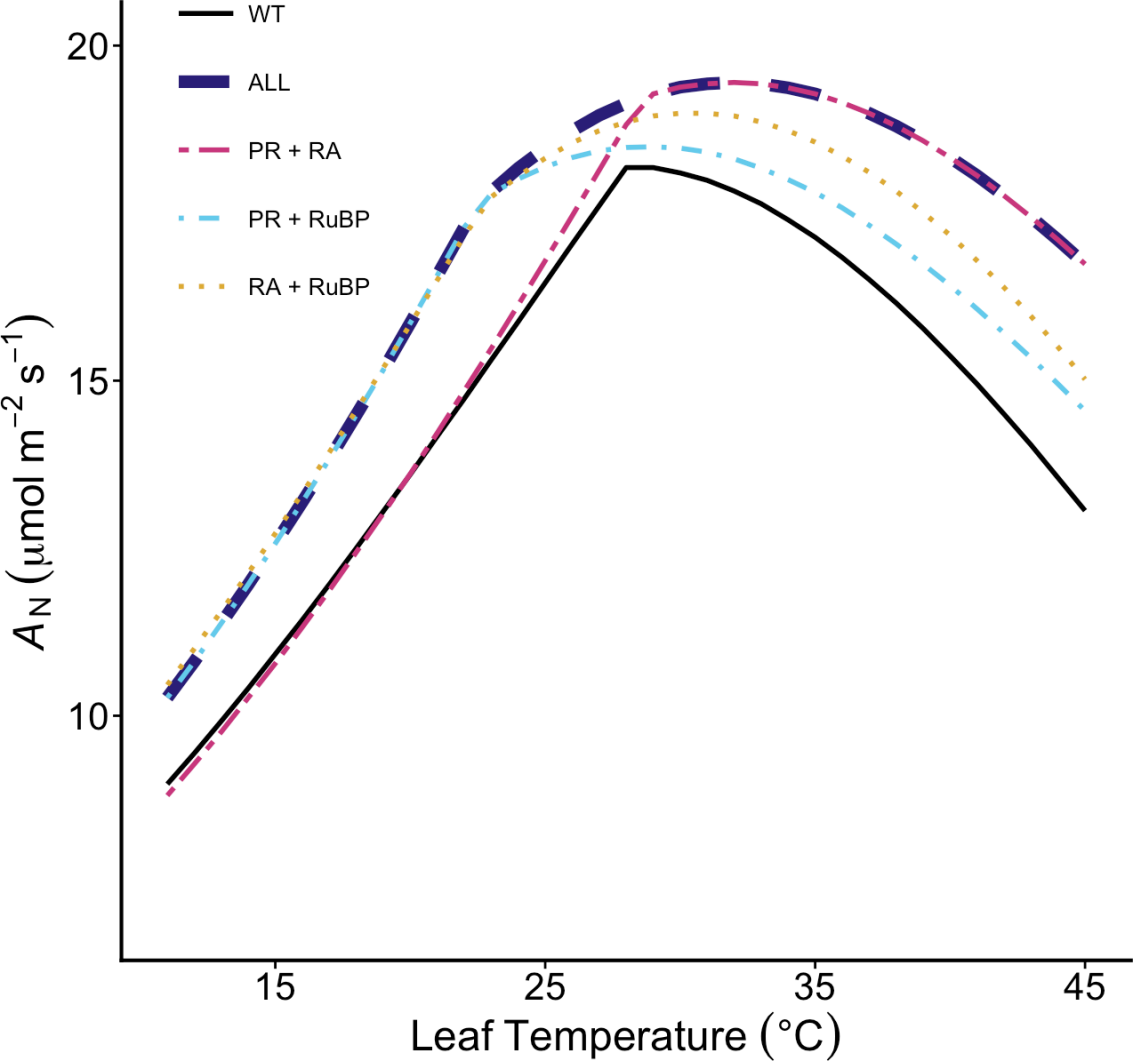
Combinatorial approaches enhance photosynthetic assimilation at and above the thermal optima. The temperature response of net photosynthetic CO_2_ assimilation rate (*A*_N_) predicted as the minimum of carboxylation-limited photosynthesis (*A*_C_) or RuBP regenerated photosynthesis (*A*_j_) based on chloroplast electron transport. Modelled outputs were obtained at 21% O_2_ and light-saturated conditions (i.e. 1500 μmol m^−2^s^−1^) assuming an ambient CO_2_ concentration of 410 ppm using parameters described in table 2 for WT (black solid line) and combinations of PR+RA (dashed pink lines), PR+RuBP (dot-dash turquoise lines), RA+RuBP (two-dash pink lines), RA+RuBP (dotted yellow lines) and all manipulations combined (thick dashed blue line).

The demonstrated impact that enhanced RA will have on *A*_N_ above the thermal optimum is aligned with recent species-independent models suggesting that the global decline in *A*_N_ can be accounted for by rubisco deactivation or declines in electron transport [16]. Our modelled responses of enhanced RA (figure 1A) support the position that PR does not limit photosynthetic carbon assimilation as much as rubisco deactivation or declines in electron transport rate, such that a more thermotolerant Rca would lead to an overall increase in photosynthesis at supra-optimal temperatures [16]. However, rubisco deactivation above the thermal optimum could act as a fine-tune control for flux into the photorespiratory pathway at elevated temperatures. Indeed, recent evidence suggests that PR enzymes downstream of rubisco do not acclimate to changes in growth temperature, such that the capacity of photorespiratory flux is scaled to rubisco activity [80]. This highlights a key consideration for improvement strategies, as enhanced RA at high temperature will increase both rubisco oxygenation and carboxylation rates, and increased metabolic flux through the photorespiratory pathway [69,81,82]. Therefore, we examined the impact of combined engineering strategies on the temperature response of photosynthesis (figure 2). When improvement strategies are combined, such that both rubisco deactivation and photorespiratory losses are minimized, modelling reveals an additive benefit to *A*_N_ at higher leaf temperatures (figure 2). At 35°C, predictions of increases relative to controls are approximately 4% when PR is modified, and approximately 8% when RA state is maintained, in line with empirical evidence from transgenic plants expressing these strategies [19,37]. Combining benefits from both strategies increases *A*_N_ by 12% at 35°C and by 19% at 40°C (figure 2). Owing to the improvements conferred by an increase in *J*_max_ below the thermal optimum, the largest benefit to assimilation across the breadth of the temperature response is realized when all three strategies are incorporated (figure 2).

## 4. Scaling from leaf-level photosynthesis to crop yield

To simulate the impact of enhanced leaf-level photosynthetic assimilation on crop yield, FvCB model parameters (table 2) were fed into the modular soybean growth simulator Soybean-BioCro [83–85] after being adjusted for the soybean *V*_cmax_ and *J*_max_ values. Soybean-BioCro was simulated using 10 years of growing season weather data compiled as in [84] from the NOAA-ESRL SURFRAD site in Bondville, Illinois, USA (2006−2015; available from ftp://aftp.cmdl.noaa.gov/data/radiation/surfrad/Bondville_IL/). These years represent a range of environmental fluctuations, including periods of drought, and give a good representation of average conditions in the region. The average air temperature over the 10 growing seasons was 21.28°C. The lowest average for a growing season was 19.9°C and the highest was 22.5°C. Yield projections were obtained for all modification combinations under ambient and elevated temperature (i.e. a constant 5°C increase above ambient), and under ambient (410 ppm) and elevated (610 ppm) atmospheric CO_2_ concentrations. Overall, elevated temperature resulted in a 15% decrease in predicted yield at 410 ppm CO_2_ concentrations (ambient: 5.5 ± 0.9 t ha^−1^; elevated 4.67 ± 1.15 t ha^−1^) and a 12% decrease at 610 ppm CO_2_ concentrations (ambient: 7.1 ± 0.8 t ha^−1^; elevated 6.3 ± 1.3 t ha^−1^). On average, warming negates the predicted yield stimulation from elevated CO_2_, as previously demonstrated for soybean using free air concentration enrichment studies [31,86].

**Figure 3.**
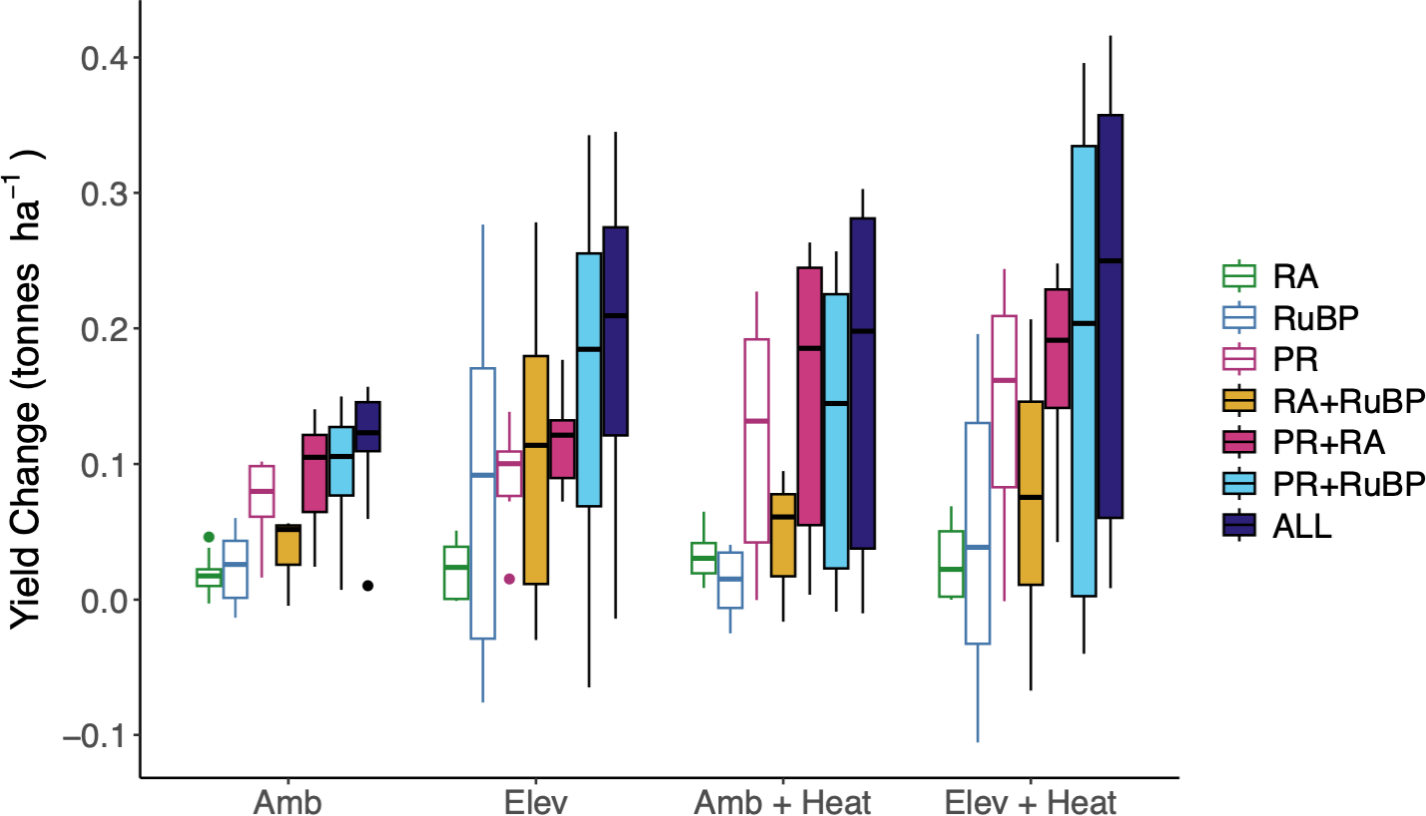
Predicted impact of photosynthetic modifications on soybean yield. Average changes in end-of-season seed yield relative to unmodified controls calculated for the years 2006−2015 growing seasons (Amb) and modelled with changes to atmospheric CO_2_ concentration (Elev; 610 ppm), and temperature (Amb + Heat; +5°C mean temperature increase), or combined climate changes (Elev + Heat; 610 ppm + 5°C mean temperature increase).

Under non-heated conditions, modifications attributed to PR confer a slight advantage to yield compared with controls in both ambient and elevated CO_2_ scenarios while those attributed to Rca offer little benefit, in line with predicted benefits to leaf-level photosynthesis at comparable temperatures (figures 2 and 3). While strategies with increased *J*_max_ (i.e. RuBP) confer the greatest predicted increase in leaf-level *A*_N_ at leaf temperatures <25°C (figure 3), this is not reflected in modelled yield increases under ambient conditions. As predicted from modelling and empirical studies [25,87], RuBP strategies confer a slight increase in yield stimulation relative to WT under elevated CO_2_ conditions, with the largest changes relative to controls at elevated CO_2_ seen in strategies combining enhanced *J*_max_ and lower photorespiratory losses. A 5°C increase in mean growing temperature lowers predicted yield overall, but the relative benefit of strategies to lower the cost of PR (PR+RA and PR+RuBP) is increased relative to ambient conditions (figure 3). However, changes attributed to RA, which demonstrate the largest benefit to light-saturated leaf-level assimilation do not confer a similarly strong predicted yield benefit under heat conditions in either ambient or elevated CO_2_ levels (figure 3). Modelled heating scenarios demonstrate large variation in yield estimates for strategies driving mean increases >1 t ha^−1^ (figure 3). This variation is largely driven by water stress, with the total rainfall for the simulated growing periods ranging from 264 to 657 mm. Strategies that increase *A*_N_ are often associated with increased stomatal conductance and subsequent water loss, which will have a more detrimental impact on yield in drier seasons. This is consistent with modelling demonstrating that increases in *A*_N_ offer limited benefit to C3 crop yield under water-limited growing conditions, despite driving yield increases under favourable conditions [88].

Scaling assimilation responses from a single leaf to a crop over a growing season adds several layers of complexity including microclimate effects throughout the canopy. Within a canopy, leaves in lower layers are shaded by overlying leaves, and typically receive only 10% of direct sunlight [89]. In crops like soybean that have dense canopies, this results in the average daytime canopy light environment being significantly lower than saturating conditions seen at the top of the canopy. To account for this, Soybean-BioCro uses a multilayer canopy model where average light is much less in bottom leaf layers compared with upper canopy layers [84,90]. In shaded conditions, the ATP : ADP ratio and redox potential in the chloroplast decline, downregulating Rca activity and leading to rubisco deactivation [54,75,91]. In these conditions, *A*_N_ is predominantly limited by electron transport and RuBP regeneration (i.e. *A*_j_; equation (2.2) , which depends on the CO_2_ compensation point Γ*) equation (2.2). Changes in the temperature response of Γ* account for enhanced thermotolerance of *A*_N_ in tobacco plants expressing modified PR [37] (table 2), which may explain the consistent yield benefit attributed to modified PR under all climate scenarios (figure 3). Although rates of rubisco oxygenation and photorespiratory flux will decrease under elevated CO_2_ scenarios, prior modelling work has also identified persistent photorespiratory-driven yield losses under elevated CO_2_ and increased temperature in wheat and soybean [69]. Therefore, lowering the cost of PR through alternative glycolate metabolism pathways [37–42] or the optimization of flux through targeted enzyme overexpression [47,50,66] remain a strategic target to improve crop yield and resilience to meet mid-century crop production goals.

Both leaf-level and crop model results assume photosynthesis is operating at a steady state. However, within the dynamic environment of the canopy, light is not constant and shaded leaves are exposed to momentary sun-flecks. These rapid periods of light induction feature changes in stromal pH, redox potential, [Mg^2+^] and [ATP], which lead to RA through Rca [92–94]. Transgenic plants overexpressing Rca exhibit faster rates of photosynthetic induction and improved growth in fluctuating light conditions [63,95,96]. Incorporating these faster induction rates would probably increase the estimated yield benefit of enhancing RA under heat stress, beyond what is captured by the crop model where only steady-state photosynthetic rates are considered (figure 3).

## 5. Conclusions: future directions and a few caveats

Models can be a powerful tool to explore photosynthetic improvement strategies under a variety of conditions in different crops before they are tested in the field. We have focused on sustained increased to atmospheric CO_2_ and growing season temperature, but future work can explore timing of heat stress and the response of changes to relative humidity, to refine mechanistic models of predicted benefits [90]. Our modelled responses are informed by physiological data gathered from a range of species, and other changes in metabolic flux associated with photosynthetic manipulation such as changes in mesophyll conductance or light induction remain unaccounted for, despite their key role in regulating carbon assimilation in a dynamic environment. Thus, the true benefits of these strategies remain to be tested under field-relevant conditions in an agronomic crop. However, the recently observed thermoprotective benefit driven by lowering photorespiratory losses in tobacco [37] and potato [44], and the yield advantage for modified field-grown soybean with enhanced RuBP regeneration under combined increases in CO_2_ and temperature [31] both suggest that our modelling outcomes are reasonable predictions. Our approach also highlights the importance of gathering mechanistic data on plants with altered photosynthetic metabolism to refine predictions of future performance. In particular, physiological temperature response data are lacking for plants overexpressing Rca or with increased RuBP regeneration capacity or electron transport rates and tends to be lacking even in non-altered metabolism plants from major crop species, complicating mechanistic modelling approaches.

Limitations to photosynthetic carbon assimilation above the thermal optimum, and strategies to mitigate these have been extensively reviewed [15,64,97–101]. Informed by empirical data from plants engineered for thermotolerance, our comparison reveals that predicted and observed benefits to leaf-level photosynthesis above the thermal optima can offer protection against yield losses owing to future warming (figure 3). Therefore, the optimization strategies discussed in this review represent strong candidates to future-proof crop yields in a changing environment.

## Data Availability

The weather data used to model crop responses is available at [102]. The code used to reproduce the results and figures in this manuscript are available in a public GitHub repository: [103].
