# Correction: ‘The heat is on: scaling improvements in photosynthetic thermal tolerance from the leaf to canopy to predict crop yields in a changing climate’ (2025), by Cavanagh and Matthews

**DOI:** 10.1098/rstb.2025.0234

**Published:** 2025-09-04

**Authors:** Amanda Cavanagh

**Affiliations:** ^1^ School of Life Sciences, University of Essex, Colchester CO4 3SQ, UK

**Keywords:** photosynthesis, temperature stress, photorespiration, climate change

*Phil. Trans. R. Soc. B* **380**, 20240235 (Published online 29 May 2025). (https://doi.org/10.1098/rstb.2024.0235)

The original published version contained an error in table 2. The transgenic temperature response parameters were incorrectly presented as scaled (normalized) values across temperatures, rather than the actual fitted parameter values used in the modelling. This correction does not affect the results or conclusions of the study. We apologize for any confusion caused.

**Table 2.** Parameters used to calculate the temperature response of *A*_N_ including the activation energy (ΔH_a_) and scaling constant (*c*). Representative values for unmodified and modified PR plants are from [37]; altered parameters are based on experimental evidence for manipulated Rca [19] (RA) and RuBP regeneration [21**,**31] (RuBP) as described in text.

	unmodified		RA		PR		RuBP	
parameter	*c*	Δ*H*_a_	*c*	Δ*H*_a_	*c*	Δ*H*_a_	*c*	Δ*H*_a_
*V*_cmax_	27.68	57.96	29.7	62.88				
*J*_max_	21.6	41.76					21.78	41.76
Γ* (Pa)	15.6	34.8			13.3	29.2		

